# How robust are the estimated effects of air pollution on health? Accounting for model uncertainty using Bayesian model averaging

**DOI:** 10.1016/j.sste.2016.04.001

**Published:** 2016-08

**Authors:** Francesca Pannullo, Duncan Lee, Eugene Waclawski, Alastair H. Leyland

**Affiliations:** a MRC|CSO Social and Public Health Science Unit, University of Glasgow, 200 Renfield Street, Glasgow, G2 3QB, UK; b School of Mathematics and Statistics, University of Glasgow, 15 University Gardens, Glasgow, G12 8QW, UK; c Public Health, University of Glasgow, 1 Lilybank Gardens, Glasgow, G12 8RZ, UK

**Keywords:** Bayesian model averaging, Conditional autoregressive models, Nitrogen dioxide, Spatial autocorrelation

## Abstract

The long-term impact of air pollution on human health can be estimated from small-area ecological studies in which the health outcome is regressed against air pollution concentrations and other covariates, such as socio-economic deprivation. Socio-economic deprivation is multi-factorial and difficult to measure, and includes aspects of income, education, and housing as well as others. However, these variables are potentially highly correlated, meaning one can either create an overall deprivation index, or use the individual characteristics, which can result in a variety of pollution-health effects. Other aspects of model choice may affect the pollution-health estimate, such as the estimation of pollution, and spatial autocorrelation model. Therefore, we propose a Bayesian model averaging approach to combine the results from multiple statistical models to produce a more robust representation of the overall pollution-health effect. We investigate the relationship between nitrogen dioxide concentrations and cardio-respiratory mortality in West Central Scotland between 2006 and 2012.

## Highlights

• We explored the sensitivity of the pollution-health effect to three factors.
• Estimation of NO2, choice of deprivation and choice of spatial autocorrelation model.
• Choice of these factors leads to a wide variation in pollution-health effects.
• BMA is utilised to estimate an overall effect while accounting for model uncertainty.
• Overall, a positive but borderline pollution-health effect was obtained.

## 1 Introduction

Air pollution has repeatedly been shown to have a detrimental impact on human health, with one of the earliest prominent examples being the London smog episode of 1952 (Health, 1954), which resulted in more than 3000 excess deaths compared with previous years. High pollution episodes such as this have lead to the implementation of air pollution legislation, such as the Clean Air Acts in 1956 and 1993 in the UK, and the 2008 Ambient Air Quality Directive in the European Union (EU). These policy practices have lead to a reduction in air pollution concentrations in many parts of the world, however a recent report by the World Health Organisation estimates that outdoor air pollution contributed to 3.7 million premature deaths in people under the age of 60 in 2012 (Organisation, 2014). Air pollution remains a serious public health problem in the UK, as nitrogen dioxide (NO_2_) concentrations currently do not meet the targets set by EU legislation. For example, in West Central Scotland, where the study presented in this paper is based, NO_2_ concentrations are predicted to exceed these targets until 2020 (Department for the Environment and Affairs, 2015).

The impacts of both long-term (chronic) and short-term (acute) exposure to pollution have been much researched, with the latter being estimated from daily ecological time-series studies (see Willocks et al., 2012). The long-term health impact of pollution is most often estimated from cohort studies (see Cesaroni et al., 2014), which make use of individual-level pollution and disease data. However, cohort studies are expensive and time consuming to implement due to the required follow-up period. This has led to spatial ecological study designs being used (see Lee, Ferguson, Mitchell, 2009, Maheswaran, Haining, Brindley, Law, Pearson, Fryers, et al., 2005), which make use of routinely available small-area data such as from the Scottish Neighbourhood Statistics (http://www.sns.gov.uk/) database, and the Health and Social Care Information Centre (http://www.hscic.gov.uk/). Due to their ecological nature these studies cannot be used to determine individual-level causality, but they contribute to and independently corroborate the body of evidence provided by cohort studies.

Spatial ecological studies are based on partitioning the study region into *n* contiguous small areas determined by administrative boundaries, such as electoral wards or census tracts. For each small area the response is the number of disease cases in a fixed time period, such as the number of deaths due to respiratory disease in one year. These disease cases are adjusted for varying population demographics across the study region using indirect standardisation, and then regressed against air pollution concentrations and other confounders, such as socio-economic deprivation. Typically, Poisson log-linear models are used to estimate the pollution-health effect, and any residual spatial autocorrelation in the data is accounted for by introducing a set of spatially autocorrelated random effects into the model. This residual spatial autocorrelation could be due to numerous factors, including unmeasured confounding (where an important spatially correlated variable is not included in the model or is unknown), neighbourhood effects (where the behaviour of subjects is influenced by surrounding subjects), and grouping effects (where subjects of similar characteristics group together). The health impact of numerous pollutants have been estimated in these studies, such as particulate matter (PM_2.5_, Lawson et al., 2012 and PM_10_, Rushworth et al., 2014), ozone (O_3_, Wang et al., 2009), sulphur dioxide (SO_2_, Elliott et al., 2007), and nitrogen dioxide (NO_2_, Maheswaran et al., 2012). In this study, we focus on NO_2_, since it is a good marker for traffic-related pollution and because measured NO_2_ data, via diffusion tubes which only measure NO_2_, are more spatially prevalent across our study region than for other pollutants.

As with all statistical modelling endeavours, estimating the effects of air pollution on health requires a number of modelling choices to be made, which are likely to affect the results. This variation in effect estimates due to model uncertainty is typically ignored, as results from a single ‘final’ model are often presented. However, it is likely to be crucial in this context because the estimated effect sizes are small and their significance will depend on the final model chosen (this is highlighted in Table 2), thus it is likely that statistically significant or non-significant results could be presented depending on the choices made by the investigators.

In this paper we investigate the impact of three such modelling choices, namely estimation of NO_2_ concentrations, the measure of socio-economic deprivation used, and the method of control for residual spatial autocorrelation. Pollution concentrations have been estimated in small-area studies using atmospheric dispersion models (see Haining, Li, Maheswaran, Blangiardo, Law, Best, Richardson, 2010, Lee, Ferguson, Mitchell, 2009), since they provide complete spatial coverage of the study region. However, these modelled concentrations are known to contain biases, and thus statistical fusion models (Berrocal, Gelfand, Holland, 2010, Fuentes, Raftery, 2005, McMillan, Holland, Morara, Feng, 2009) are increasingly being used, which calibrate the modelled concentrations with observed pollution measurements. Socio-economic deprivation is the major confounder in these studies, and existing studies have either used individual variables such as job seekers allowance or house price (Lee et al., 2014) or composite indexes such as the Townsend index (Maheswaran et al., 2005) to account for it. Finally, residual spatial autocorrelation can be ignored by fitting a simple Poisson log-linear model to the data, while a common adjustment is to add a set of random effects represented by a conditional autoregressive (CAR, Besag et al., 1991) prior to the linear predictor. However, Clayton et al. (1993) and Paciorek (2010) have shown this may lead to collinearity between the fixed and random effects, and a number of extensions have been proposed such as Hughes and Haran (2013) and Lee and Sarran (2015).

Therefore, in this paper we present a new study of NO_2_ concentrations and cardio-respiratory mortalities in West Central Scotland, in which we quantify empirically how robust the estimated pollution-health effect sizes are to these factors, and present a Bayesian model averaging (BMA, Hoeting, Madigan, Raftery, Volinsky, 1999, Raftery, 1995) approach to estimating the overall effect size whilst accounting for model uncertainty. Section 2 describes our motivating study, along with descriptions of the disease, air pollution and deprivation data. Section 3 presents the statistical models described above for taking into account residual spatial autocorrelation and estimating NO_2_ concentrations. This section also presents the BMA methodology for combining the estimated air pollution effects from the range of models considered. Our results from the individual models and BMA are presented in Section 4, while Section 5 provides a concluding discussion.

## 2 Motivating study

**Fig. 1:**
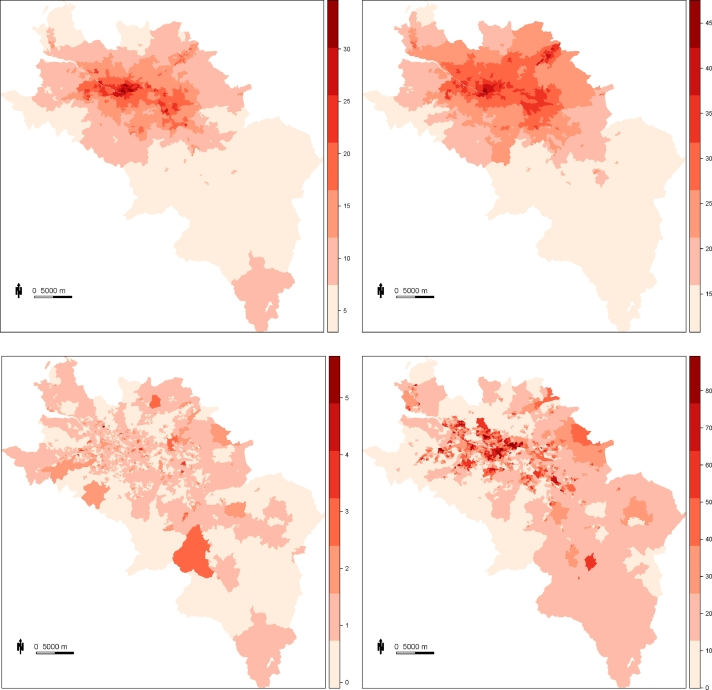
Display of the data. The top left panel shows background NO_2_ concentrations provided by DEFRA from an atmospheric dispersion model averaged across 2006–2012, while the top right panel shows estimates from a statistical fusion model. The bottom left panel displays the Standardised mortality ratio (SMR) for cardio-respiratory disease aggregated over 2006–2012, while the bottom right panel displays the SIMD score (without health domain), where a high score indicates deprivation and a low score indicates affluence.

The methodology developed in this paper is motivated by a new epidemiological study investigating the health impact of long-term exposure to air pollution in West Central Scotland, for the seven year period 2006 to 2012. West Central Scotland is centred around the Greater Glasgow conurbation, which has a population of around 1.1 million people and a land area of 386 km^2^. West Central Scotland is partitioned into n=2089 non-overlapping data zones, which are the key small area geography in Scotland comprising between 500 and 1000 residents of similar social characteristics. These data zones are described at the Scottish Neighbourhood statistics website (SNS, http://www.sns.gov.uk/). The layout of the study region is presented in Fig. 1, where the city of Glasgow is the set of small data zones in the middle north of the figure.

### 2.1 Description of the data

The disease data comprise counts of the numbers of cardio-respiratory mortalities (International Classification of Diseases, 10th Revision: I00-I99, J00-J99) within each of the 2089 data zones during the seven year period 2006 to 2012. These death records were obtained from National Records Scotland. In order to take into account the heterogeneity of the population within each data zone in terms of their size and demographic structure, the expected numbers of cardio-respiratory mortalities were calculated by indirect standardisation, using age- and sex-specific cardio-respiratory mortality rates for the whole of West Central Scotland. However, due to the low numbers of cardio-respiratory mortalities occurring in a single year (mean of 4.093 for 2006), the cardio-respiratory deaths have been aggregated across the seven year period. The spatial distribution of disease risk is shown in the bottom left panel of Fig. 1, which displays the standardised mortality ratios (SMR, observed numbers/expected numbers) across West Central Scotland for the aggregated years 2006 to 2012. A SMR of 1.2 corresponds to a 20% increase in the risk of disease compared to what is expected. The highest SMRs are found in areas with the highest level of deprivation, and range between 0 and 4.747, with a mean SMR of 1.066, and a standard deviation of 0.440. Data zones have a SMR of zero when there have been no deaths, which occurs mostly in the centre of Glasgow as it consists mainly of shopping districts.

Concentrations of NO_2_ are available from two sources: measured data from automatic monitors and diffusion tubes, and modelled concentrations from an atmospheric dispersion model. The set of measured data are more spatially dense than corresponding data for other pollutants (mainly thanks to the inclusion of diffusion tubes), but still do not give complete spatial coverage of all 2089 data zones in West Central Scotland. Therefore the simplest approach is to only use the modelled concentrations, which are annual mean background concentrations available at a 1km × 1km resolution for the seven year period from the Department for Environment, Food and Rural Affairs (DEFRA, http://uk-air.defra.gov.uk/). These concentrations are temporally aggregated over the seven years by averaging, and are spatially aggregated to the data zone level using

(1) $$\begin{array}{c} \text{NO}{}_{2_{i}}=\frac{\sum_{j=1}^{n_{i}}\exp\left(-d_{ij}\right){\tilde{{\text{NO}}_{2}}}_{j}}{\sum_{j=1}^{n_{i}}\exp\left(-d_{ij}\right)}, \end{array}$$

where NO${}_{2_{i}}$ is the averaged concentration for data zone *i*. Here ${\tilde{{\text{NO}}_{2}}}_{1},\ldots ,{\tilde{{\text{NO}}_{2}}}_{n_{i}}$ are the modelled NO_2_ concentrations at the *n_i_* grid squares within data zone *i*, and *d_ij_* is the Euclidean distance between the population weighted centroid of data zone *i* and the centroid of grid square *j*. This is to ensure that the data zone takes a representative value according to the location at which the population density is greatest. Data zones containing no grid square centroids were assigned the NO_2_ concentration nearest the population weighted centroid of the data zone. These data zone averaged modelled concentrations are displayed in the top left panel of Fig. 1, and the city of Glasgow has the highest level of background concentrations, as expected. However, these modelled concentrations are known to contain biases, and in this paper we compare the health effects estimated from using them to those obtained from predicting NO_2_ with a statistical fusion model. A number of statistical fusion models have been proposed for combining measured and modelled pollution data, including Berrocal et al. (2010); Fuentes and Raftery (2005); McMillan et al. (2009). However, the model we use here is that of Pannullo et al. (2015), which uses a similar approach to that of Berrocal et al. (2010) and was developed for the West Central Scotland study region. The general form of the model is given by

(2) $$\begin{array}{ccc} & & V\left(s_{k}\right)∼\text{N}\left(b{\left(s_{k}\right)}^{⊤}\alpha +\phi \left(s_{k}\right),\nu^{2}\sigma^{2}\right),\;k=1,\ldots ,m,\\ & & \phi =\left(\phi \left(s_{1}\right),\ldots ,\phi \left(s_{m}\right)\right)∼\text{N}\left(0,\sigma^{2}\Sigma \left(\rho \right)\right), \end{array}$$

where *V*(**s**_*k*_) is the measured NO_2_ concentration at spatial location **s**_*i*_ for i=1,…,m spatial locations. The *m* measurements are modelled by a set of covariates **b**(**s**_*k*_) with regression parameters α, and the former include the modelled concentration in the nearest grid square and the local environment in which the site is located (e.g. roadside, urban background, rural). The second term in the mean model is a spatial random effect $\phi =(\phi \left(s_{1}\right),\ldots ,\phi \left(s_{m}\right)),$ which accounts for residual spatial autocorrelation in the measured data and are modelled by a Gaussian process with a spatial exponential correlation matrix Σ(ρ) where *ρ* denotes the range parameter. Full details of this model are available from Pannullo et al. (2015).

This model calibrates the modelled concentrations according to urban background and rural environments since the West Central Scotland study region has a large proportion of rural areas, while allowing the effect of the modelled concentrations to vary across space. Pannullo et al. (2015) showed that their fusion model produced improved predictions of the measured data in a cross-validation exercise compared to using the modelled concentrations in isolation, and predictions were made from the model on a 1 km × 1 km resolution for each year separately. These predictions were then temporally aggregated by averaging and spatially aggregated up to data zone level using the same form as (1). The resulting concentrations are displayed in the top right panel of Fig. 1, which is structurally similar to the modelled concentrations as expected.

The main confounding factor in ecological health studies is socio-economic deprivation (Mackenbach et al., 1997), in which populations with higher levels of deprivation may be more susceptible to the effects of air pollution (Laurent et al., 2007), for example due to them having worse underlying health on average compared with more affluent communities. However, deprivation is multi-factorial and difficult to measure, and is commonly represented by a composite index. Here we make use of the Scottish Index of Multiple Deprivation (SIMD, http://www.gov.scot/Topics/Statistics/SIMD), which is a composite index containing seven domains, namely: access to services; crime; education, skills and training; employment; income; health; and housing. The health domain is not used in this study since it contains an indicator of the Comparative Mortality Factor, and therefore includes deaths which are part of the outcome in our study. Correlations between the remaining six domains are displayed in Table 1, where there are high correlations between income, employment, and education; and weak to moderate correlations with the access, housing and crime domains. The overall SIMD index is not appropriate for this study since it contains the health domain. Therefore, we re-weighted the overall index to remove the health domain based on the original index methodology, details of which can be found here http://www.gov.scot/Publications/2004/10/20089/45173. The bottom left panel of Fig. 1 displays the re-weighted overall index, where it is clear that the city of Glasgow contains the majority of the most deprived areas, as expected.

Initially, a simple Poisson log-linear model (without any spatial random effects) was fitted to the data with NO_2_ (DEFRA modelled concentrations) and income deprivation as covariates, and the overdispersion parameter was estimated as 4.35, suggesting substantial overdispersion with respect to the Poisson assumption of equal mean and variance. The residuals from this model were then tested for spatial autocorrelation, using a permutation test based on Moran’s I statistic (Moran, 1950). The null hypothesis of this test is no spatial autocorrelation, and Moran’s I statistic was 0.036 with a p-value of 0.003, suggesting that spatial autocorrelation is present in the residuals.

## 3 Statistical models for estimating air pollution and health effects

The aim of this paper is to estimate the sensitivity of the estimated relationship between NO_2_ concentrations and cardio-respiratory mortality in the West Central Scotland region between 2006 and 2012. In doing this we estimate the sensitivity of the effect to changing the estimation of NO_2_ concentrations, control for socio-economic deprivation, and allowance for residual spatial autocorrelation in the mortality data after adjusting for the covariate effects. We compare three specific Poisson log-linear models in this sensitivity analysis, which differ in their control for residual spatial autocorrelation. These models are then combined to estimate an overall pollution-health effect using Bayesian model averaging, with inference based on Markov chain Monte Carlo (McMC) simulation. These models are implemented in the R software environment (R Core Team, 2015), using the CARBayes (Lee, 2013) and ngspatial (Hughes and Cui, 2015) packages, while Bayesian model averaging was implemented using code written by the authors. Sensitivity to the estimation of NO_2_ and control for socio-economic deprivation is assessed by fitting different covariate combinations in all of the three models described below.

### 3.1 Data and likelihood model

The vector of the observed numbers of cardio-respiratory mortalities is denoted by $Y=(Y_{1},\ldots ,Y_{n}),$ while the expected numbers of mortalities are computed using indirect standardisation based on age- and sex-specific cardio-respiratory mortality rates in West Central Scotland. These expected counts are denoted by $E=(E_{1},\ldots ,E_{n}),$ where for data zone *i*, $E_{i}=\sum_{r}N_{ir}\gamma_{r},$ where *N_ir_* is the number of people in age-sex group *r* in data zone *i*, and *γ_r_* denotes the region-wide age-sex mortality rate. The vector of NO_2_ concentrations is denoted by $x=(x_{1},\ldots ,x_{n})$ for all *n* data zones, while each measure of socio-economic deprivation is denoted by $u=(u_{1},\ldots ,u_{n})$. Thus, for the *i*th data zone the vector of covariates is given by $z_{i}^{⊤}=\left(1,x_{i},u_{i}\right)$ while the corresponding parameters are given by $\beta =(\beta_{1},\beta_{2},\beta_{3}),$ so that *β*_1_ is the intercept term while *β*_2_ is the key parameter in this model, namely the effect of NO_2_ on cardio-respiratory mortality risk. A general Bayesian Poisson log-linear model for these data is given by

(3) $$\begin{array}{ccc} & & Y_{i}|E_{i},R_{i}∼\text{Poisson}\left(E_{i}R_{i}\right)\;\text{for}\;i=1,\ldots ,n,\\ & & \ln\left(R_{i}\right)=z_{i}^{⊤}\beta +\phi_{i},\\ & & \beta ∼\text{N}(m,V), \end{array}$$

where *R_i_* is the risk of disease in data zone *i*. The regression parameters β are assigned a weakly informative multivariate Gaussian prior with hyperparameters (**m, V**), typically with mean zero and a large diagonal variance matrix. The final term in the linear predictor $\phi =(\phi_{1},\ldots ,\phi_{n})$ controls for the residual spatial autocorrelation in the data after accounting for the covariate effects, and we consider three modelling specifications here.

### 3.2 Model 1 – no spatial autocorrelation

The simplest approach is to ignore the presence of any residual spatial autocorrelation and assume $\phi_{i}=0$ for all data zones *i*, which is equivalent to fitting a Poisson generalised linear model to the data. This model naively assumes the cardio-respiratory counts are independent conditional on the covariates, which as illustrated in Section 2 is not true for our case study. Additionally, the model does not allow for overdispersion relative to the Poisson likelihood, and thus makes the restrictive assumption that $E\left[Y_{i}\right]=\mathrm{Var}\left[Y_{i}\right],$ which was shown in Section 2 to be unrealistic. This model is thus included here for comparison purposes with the other models described below.

### 3.3 Model 2 – globally smooth spatial autocorrelation

The standard approach to accounting for residual spatial autocorrelation and overdispersion in this context is to model ϕ by a set of globally spatially smooth (autocorrelated) random effects $\phi =(\phi_{1},\ldots ,\phi_{n})$. A number of models can be specified for these random effects, including conditional autoregressive (CAR), simultaneous autoregressive (SAR) or geostatistical models. However, CAR priors are the most common in this field, and examples of their use include Maheswaran et al. (2005) and Lee et al. (2009). A number of globally smooth CAR priors have been proposed, and a review by Lee (2011) concluded that the model proposed by Leroux et al. (1999) was the most appealing. This model can be specified by a set of *n* univariate full conditional distributions $f(\phi_{i}|\phi_{-i}),$ where $\phi_{-i}=\left(\phi_{1},\ldots ,\phi_{i-1},\phi_{i+1},\ldots ,\phi_{n}\right)$. Spatial autocorrelation is imposed using a binary *n* × *n* neighbourhood matrix **W**, whose *ij*th element $w_{ij}=1$ if areas (*i, j*) share a common border, and $w_{ij}=0$ otherwise. This specification asserts that neighbouring areas have random effects that are partially autocorrelated, otherwise the random effects are conditionally independent. The model has the form

(4) $$\begin{array}{c} \phi_{i}|\phi_{-i}∼\text{N}\left(\;\frac{\rho \sum_{j=1}^{n}w_{ij}\phi_{j}}{\rho \sum_{j=1}^{n}w_{ij}+1-\rho },\frac{\tau^{2}}{\rho \sum_{j=1}^{n}w_{ij}+1-\rho }\;\;\right), \end{array}$$

where *ρ* controls the level of spatial autocorrelation, with ρ=0 corresponding to spatial independence with mean zero and constant variance, and ρ=1 corresponding to strong spatial autocorrelation (and simplifying to the intrinsic CAR model). Weakly informative hyperpriors are assigned for *τ*^2^ and *ρ*; typically an inverse-gamma(*a, b*) distribution for *τ*^2^, and a uniform distribution on the unit interval for *ρ*.

### 3.4 Model 3 – orthogonal smoothing

One problem with traditional CAR models such as (4), is that the spatially smooth random effects have been shown to be correlated with the spatially smooth fixed effects, such as air pollution (Paciorek, 2010). This spatial confounding between the fixed and random effects leads to variance inflation and the model parameters becoming uninterpretable. Much research has been conducted on controlling for this spatial confounding, in which the random effects are instead modelled with a series of basis functions that are orthogonal to the covariates, thus mitigating this confounding (Hughes, Haran, 2013, Reich, Hodges, Zadnik, 2006). In this paper, we utilise the orthogonal smoothing model proposed by Hughes and Haran (2013) due to the low dimensionality of the random effects, which leads to fast computation. This model replaces the random effects *ϕ_i_* in Eq. (3) with a linear combination of basis functions that are orthogonal to the fixed effects. Let the matrix of *p* covariates be denoted by $Z={\left(z_{1}^{⊤},\ldots ,z_{n}^{⊤}\right)}^{⊤},$ then the orthogonal projection matrix (hat matrix) onto the column space of the design matrix **Z** is defined by

(5) $$\begin{array}{c} P=Z{\left(Z^{⊤}Z\right)}^{-1}Z^{⊤}, \end{array}$$

and let the residual projection matrix onto the space orthogonal to **Z** be defined by

(6) $$\begin{array}{c} P^{\prime }=I_{n}-P. \end{array}$$

The residual projection matrix is then used to create a set of eigenvectors, from the matrix product **P**′**WP**′, which combines covariate orthogonality given by **P**′ with spatial adjacency given by **W**. The eigenvectors of **P**′**WP**′ contain all possible mutually distinct spatial patterns of clustering orthogonal to **Z**. Furthermore, spatial dependence is related to both positive and negative eigenvalues, where positive eigenvalues correspond to positive spatial autocorrelation. The size of the eigenvalue associated with a given eigenvector determines the relative importance of its spatial pattern, so Hughes and Haran (2013) suggest only selecting the first *q* < <*n* eigenvectors corresponding to the largest positive eigenvectors. This matrix is denoted by **M**, where $m_{i}^{⊤}=\left(m_{i1},\ldots ,m_{iq}\right)$. The chosen number of eigenvectors *q* acts as a tuning parameter, which determines the extent of dimensionality reduction in the model, and the authors suggest using q=50 as a default choice. The orthogonal smoothing model replaces the random effects in the linear predictor in Eq. (3) by

(7) $$\begin{array}{ccc} & & \ln\left(R_{i}\right)=z_{i}^{⊤}\beta +m_{i}^{⊤}\delta ,\\ & & \delta ∼\text{N}(0,\tau^{2}Q{\left(W\right)}_{s}^{-1}), \end{array}$$

where the random effects δ are assigned a Gaussian prior with mean **0**, and precision matrix given by $Q{\left(W\right)}_{s}=M^{T}Q\left(W\right)M,$ where Q(W)=diag(W1)−W corresponds to the precision matrix for the intrinsic CAR prior (Besag et al., 1991).

### 3.5 Bayesian model averaging

Bayesian model averaging provides a coherent framework for combining the estimates of the same quantity of interest from a number of different Bayesian models into a single overall estimate, which accounts for model uncertainty. Such model uncertainty is often ignored in existing studies, and as we show in the next section can have a large impact on the results. Recall that *β*_2_ is the key parameter of interest in this model, namely the effect of NO_2_ concentrations on cardio-respiratory mortality risk. Consider the case of having *K* candidate models, where in our study we have K=42 (see next section for details). Denote these models by $\left(M_{1},\ldots ,M_{K}\right)$ and their respective sets of model parameters by $\left(\theta_{1},\ldots ,\theta_{K}\right)$. Let *β*_2_ denote the true unknown parameter of interest and ${\hat{\beta }}_{2_{k}}$ denote the estimate (posterior median) from the *k*th model. Then the posterior distribution of interest is

(8) $$\begin{array}{c} f\left(\beta_{2}|Y\right)=\sum_{k=1}^{K}f\left(\beta_{2}|M_{k},Y\right)f\left(M_{k}|Y\right). \end{array}$$

Here *f*(*β*_2_ | *M_k_*, **Y**) is the posterior distribution of *β*_2_ from model *K*, and *f*(*M_k_* | **Y**) is the posterior probability of model *M_k_*. This equation essentially averages the posterior distributions for *β*_2_ under each model weighted by their posterior model probabilities. The posterior probability for model *M_k_* is given by

(9) $$\begin{array}{c} f\left(M_{k}|Y\right)=\frac{f\left(Y|M_{k}\right)f\left(M_{k}\right)}{\sum_{l=1}^{K}f\left(Y|M_{l}\right)f\left(M_{l}\right)}, \end{array}$$

where *f*(*M_k_*) is the prior probability for model *M_k_*. We specify our prior ignorance via a discrete uniform prior for *f*(*M_k_*), that is $f(M_{k})=1/K$. This specification simplifies the posterior probability for model *M_k_* in Eq. (9) to

(10) $$\begin{array}{c} f\left(M_{k}|Y\right)=\frac{f(Y|M_{k})}{\sum_{l=1}^{K}f\left(Y|M_{l}\right)}. \end{array}$$

The marginal (averaged over the parameters) probability of the data given model *M_k_* is computed by

(11) $$\begin{array}{c} f\left(Y|M_{k}\right)=\int_{\theta_{k}}f\left(Y|\theta_{k},M_{k}\right)f\left(\theta_{k}|M_{k}\right)d\theta_{k}, \end{array}$$

which can be approximated by *J* McMC samples as

(12) $$\begin{array}{c} f\left(Y|M_{k}\right)\approx \frac{1}{J}\sum_{j=1}^{J}f\left(Y|\theta_{k}^{\left(j\right)},M_{k}\right)f\left(\theta_{k}^{\left(j\right)}|M_{k}\right), \end{array}$$

where $\theta_{k}^{\left(j\right)}$ is the *j*th McMC sample for model *M_k_*. Once these quantities have been computed the posterior mean and variance of *β*_2_ are given by:

(13) $$\begin{array}{ccc} E(\beta_{2}|Y) & = & \sum_{k=1}^{K}{\hat{\beta }}_{2_{k}}f\left(M_{k}|Y\right), \end{array}$$

(14) $$\begin{array}{ccc} \text{Var}(\beta_{2}|Y) & = & \sum_{k=1}^{K}\left[\text{Var}\left(\beta_{2}|M_{k},Y\right)+{\hat{\beta }}_{2_{k}}^{2}\right]f\left(M_{k}|Y\right)\\ & & -\;E{\left(\beta_{2}|Y\right)}^{2}, \end{array}$$

where Var(*β*_2_ | *M_k_*, **Y**) is the posterior variance of *β*_2_ from model *M_k_*. Based on a normal approximation to the posterior, an approximate 95% credible interval can be obtained for *β*_2_ that accounts for model uncertainty.

## 4 Results from the West Central Scotland study

We now present the results of our study investigating the long-term effects of NO_2_ concentrations on cardio-respiratory mortality in West Central Scotland between 2006 and 2012. Section 4.1 describes the set of results obtained from fitting the range of models described in this paper, which illustrates the sensitivity of the results due to model choice. Then Section 4.2 presents our overall estimate of the effect of NO_2_ on cardio-respiratory mortality using the Bayesian model averaging approach outlined in the previous section. Inference for all models described in this section is based on running 5 parallel Markov chains for 120,000 iterations, which included a burn-in period of 20,000 iterations. The remaining samples were thinned by 10 to reduce their autocorrelation, thus producing a final set of 50,000 posterior samples across the five chains.

### 4.1 Results – sensitivity to model choice

We empirically investigate the sensitivity of the estimated pollution-health effect to three modelling choices. The first is the estimation of spatially averaged NO_2_ concentrations for each data zone, and we compare averaging the raw output from the atmospheric dispersion model used by DEFRA (http://uk-air.defra.gov.uk/, denoted *DEFRA*) to averaging predictions from the fusion model proposed by Pannullo et al. (2015) (denoted *Fusion*). The second modelling choice is how to control for the confounding effects of socio-economic deprivation, and we compare using the composite Scottish Index of Multiple Deprivation (SIMD, minus the health domain), with individual indicators from its sub-domains, namely access to services, crime, education, employment, housing and income. Finally, we compare three approaches to controlling for residual spatial autocorrelation, ignoring it (denoted *GLM*), modelling it using random effects represented by the globally smooth model proposed by Leroux et al. (1999) (denoted *Leroux*), and modelling it using a set of orthogonal random effects proposed by Hughes and Haran (2013) (denoted *OS*).

All combinations of these factors gives a set of 42 possible models, and the results are summarised in Table 2 and 3, which respectively display the posterior median relative risks and 95% credible intervals, and the Deviance Information Criterion (DIC, Spiegelhalter et al., 2002) together with the effective number of parameters (*p_D_*) for each model and the root mean square error (RMSE) of the data **Y**. All pollution-health effects are presented on the relative risk scale for a 5 *μ*gm${}^{-3}$ increase in NO_2_ concentrations (for both *DEFRA* and *Fusion*), as this is a realistic change in long-term exposure. Overall, there is evidence that increasing NO_2_ concentrations is associated with small but positive increases in the risk of cardio-respiratory mortality, as 36 out of the 42 models estimate the relative risk to be greater than 1. However, the range of the effects estimated across the 42 models is large, being between a 2% decreased risk (0.980) to a 5.3% increased risk (1.053) associated with a 5 *μ*gm${}^{-3}$ increase in NO_2_. This suggests that the results are highly sensitive to model choice, and that if we had presented results from a single model then we could have shown either a positive or a negative effect of NO_2_ on mortality risk. Focusing on the 95% credible intervals shows that 23 of the 42 intervals are wholly above the null risk of 1, which is just over 57% of the models considered.

The three modelling choices considered here all appear to have the potential to substantially effect the estimated relative risks, as the estimates from varying one factor at a time can lead to large changes in risk. For example, changing the NO_2_ metric from that produced by the fusion model to that produced by DEFRA resulted in the risks changing by between −1% and 5.3%, and in all but 3 cases these changes were positive. This indicates that overall using the DEFRA concentrations resulted in increased risks compared with using the predictions from the fusion model. Changing the control for socio-economic deprivation also had a large impact on the results, with changes in relative risk of between 3.8% and 7.3% across the 7 measures considered depending on the combination of *DEFRA/Fusion* and *GLM/Leroux/OS*. In general, using the housing indicator resulted in the lowest effect sizes, while using crime resulted in the highest estimates. Finally, varying the control for spatial autocorrelation had a slight effect on the results, with differences in relative risk between the three models considered ranging between 0.1% and 0.9%. The only pattern of note is that the effect sizes are attenuated for the *OS* models compared to the *Leroux* models in 10 out of the 14 models, with 2 models comprising the same effect size.

Finally, Table 3 summarises the fit of each model via the DIC and RMSE, which shows that in all cases the *Leroux* model fits the data best compared with the other alternatives. This is surprising considering the globally smooth model has the potential for correlation between the fixed and random effects and thus one would expect the *OS* model to outperform the *Leroux* model. The *OS* model has many fewer effective number of parameters compared to the *Leroux* model, which makes it more parsimonious. However, it is this reduction in dimensionality that has resulted in a poorer fit to the data (in terms of DIC). In most cases, the DIC is lower for the *DEFRA* concentrations compared to the *Fusion* concentrations, while the income domain provides the best fit to the data of all the socio-economic indicators considered here. Furthermore, the RMSE allows us to assess the closeness of the models fitted values to the observed health outcomes (with the lowest values indicating better performance), and to give a sense of scale the 25th and 75th percentiles of the observed cardio-respiratory deaths were 14 and 33 respectively. The *Leroux* models have the lowest RMSE values compared to the *GLM*s and *OS* models, which is due to their increased number of effective parameters. For the *Leroux* models the relationship between RMSE and deprivation is opposite to that observed for the *GLM*s and *OS* models, with income, the best deprivation covariate in terms of DIC, having the highest RMSE compared to the other deprivation measures (2.693 compared to 2.518 for access). The reason is that after adjusting for income deprivation there is less residual spatial variation in the model compared to using the other deprivation covariates. Thus, the random effects have less spatial variation, and thus less impact on the fitted values. For example, the variance *τ*^2^ is 0.230 for the access covariate compared to 0.094 for income. This is also observed in the effective number of parameters *p_D_*, which is smallest for the model with income. In contrast, the DIC is an overall measure of model quality that penalises more complex models containing more parameters, hence higher DIC values for access (and others) compared to income.

### 4.2 Results – BMA

The previous section shows clear sensitivity of the results to the model fitted, and one solution would be to choose a single ‘best’ model, for example by minimising the DIC. However this clearly ignores model uncertainty, which can be accounted for using BMA as described in Section 3.5 by combining the estimated effect sizes from the 42 models considered here. When this was done the overall estimated relative risk was 1.011 together with associated 95% uncertainty interval of (0.993, 1.029). This small but positive effect indicates that for a 5 *μ*gm${}^{-3}$ increase in NO_2_ concentrations cardio-respiratory deaths increase by an estimated 1.1%, although the lower end of the 95% credible interval is below the null risk of 1. In fact, the posterior probability that the relative risk is greater than 1 is 0.884. This result is essentially a mixture between the effect estimates from the *Leroux* model including income and *DEFRA* NO_2_ concentrations, and the effect estimate from the *Leroux* model including income and *Fusion* model NO_2_ concentrations. The former had the most influence on the overall effect size since its posterior model probability *f*(*M_k_*| **Y**) was 67.82%, whilst it was 32.17% for the latter. So in this example the large differences in fit across the 42 models has resulted in only two models contributing to the overall effect estimate.

## 5 Discussion

In this paper we investigated the sensitivity of the pollution-health relationship in West Central Scotland to the impact of three modelling choices: the estimation of NO_2_ concentrations, control for socio-economic deprivation, and control for residual spatial autocorrelation after accounting for the covariate effects. Our main finding is that the choice of these three factors can have a major impact on the resulting pollution-health effects, which means that if only a single model was presented researchers could show a wide range of effect sizes depending on which model is chosen. The estimated pollution-health effect in this study varies considerably across the 42 models (effect sizes range from 0.980–1.053), highlighting the estimated pollution-health effect sizes are not robust to the three aforementioned factors.

We then utilised BMA to combine the results from all 42 models into an overall pollution-health effect size, whilst taking into account model uncertainty. Our final estimated effect size shows a 5 *μ*gm${}^{-3}$ increase in NO_2_ concentrations is associated with 1.1% higher cardio-respiratory deaths in West Central Scotland between 2006 and 2012. However, this effect is (borderline) not substantial at the 5% level, as the resulting 95% credible interval contains the null risk of 1. This could be due to the fact that the majority of the NO_2_ concentrations are relatively low, and thus greater variation in the exposure would be needed to observe substantial health impacts.

A second finding from our study is the attenuation of the pollution-health effects when the NO_2_ concentrations were estimated using the geostatistical fusion model, compared to when the NO_2_ concentrations were estimated by the DEFRA diffusion model. The estimated health effects changed between −1% and 5.3%, indicating that increased risks are observed when the DEFRA concentrations are utilised. This is an interesting result considering the majority of spatial ecological studies in Scotland (and indeed in the UK) make use of modelled concentrations because of their wide availability and fine scale spatial coverage. Furthermore, the correlation between residual disease (after adjustment from income deprivation) and pollution from both the fusion and DEFRA models is 0.041 and 0.029 respectively. This highlights that the DEFRA pollution concentrations are more correlated with residual disease, thus explaining why it has a stronger effect size (see Table 2) compared to the pollution concentrations from the fusion model. However, in terms of pollution predictive performance, Pannullo et al. (2015) show that the DEFRA data are not as good at predicting measured pollution concentrations at the point level, since the root mean square prediction error (RMSPE) is 0.337 compared to 0.255 for the fusion model. Furthermore, a recent study conducted in mainland Scotland by Huang et al. (2015) concluded that the estimated health effects of NO_2_ were largely consistent when estimated from a fusion model compared to modelled concentrations from the DEFRA.

A third finding from our study is that the global spatial autocorrelation model comprising the DEFRA concentrations and income deprivation dominated the overall pollution-health effect size when combining models using BMA. The posterior model probability was 67.82%, while for the model with the fusion model concentrations it was 32.17%. It is interesting to note that only 2 out of the 42 models had a considerable influence, suggesting that the global spatial autocorrelation model with the income deprivation and DEFRA concentrations are the most important factors for investigating the impact of air pollution on health in West Central Scotland. In addition, the global spatial autocorrelation model, which has been under much scrutiny by Reich et al. (2006) and *others*, outperformed the orthogonal smoothing model proposed by Hughes and Haran (2013) in terms of model fit via the DIC.

There are a few drawbacks to our study. Firstly, we had to aggregate our cardio-respiratory deaths over a 7 year period to ensure there was enough variation in the disease data. This meant only a purely spatial study could be performed and it would be important to investigate how the pollution-health risks have changed over time. In addition, there may not be enough cardio-respiratory deaths at the data zone level, thus upgrading to a larger spatial resolution, such as intermediate geographies (average population of 4300 inhabitants), may improve the power of the study to detect an association. Furthermore, hospital admission data could be used instead as the health outcome since there will be more events and thus no need to aggregate over multiple years. Secondly, the DEFRA modelled concentrations come with no measure of uncertainty and this could impact the analysis. The predicted concentrations from the geostatistical fusion model do have measures of prediction uncertainty, however, in this study we treated the predicted NO_2_ concentrations as the known and true values, which again could impact our results. Therefore, an avenue for future work will be to incorporate the uncertainty surrounding the predicted NO_2_ concentrations in a combined Bayesian framework, which estimates the exposures and health risks simultaneously. Finally, our key message is that due to the small effect sizes, a range of pollution-health effects can be estimated depending on the modelling choices made, and thus accounting for this is vital to provide robust evidence of the damaging effect of air pollution on health.

## Figures and Tables

**Table 1:** Correlations between the six deprivation measures, where EST denotes the education, skills and training domain.

Variable	Access	Crime	EST	Employment	Income	Housing
Access	1	−0.252	−0.250	−0.287	−0.321	−0.411
Crime	–	1	0.411	0.436	0.430	0.351
EST	–	–	1	0.833	0.860	0.680
Employment	–	–	–	1	0.946	0.436
Income	–	–	–	–	1	0.658
Housing	–	–	–	–	–	1

**Table 2:** Posterior median relative risks (RR) and 95% credible intervals for a 5 *μ*gm${}^{-3}$ increase in NO_2_ concentrations on cardio-respiratory mortality. The results displayed relate to models varying in their estimation of NO_2_, control for deprivation and allowance for residual spatial autocorrelation. The results in bold are substantial effects at the 5% level.

Deprivation	Model	RR (95% CI)	
		Fusion	DEFRA
Access	GLM	**1.036 (1.016, 1.056)**	**1.050 (1.026, 1.075)**
	Leroux	**1.033 (1.006, 1.059)**	**1.045 (1.015, 1.075)**
	OS	**1.029 (1.020, 1.039)**	**1.041 (1.030, 1.051)**

Crime	GLM	**1.038 (1.018, 1.058)**	**1.053 (1.033, 1.074)**
	Leroux	**1.039 (1.015, 1.063)**	**1.053 (1.027, 1.079)**
	OS	**1.034 (1.025, 1.043)**	**1.046 (1.037, 1.057)**

Education	GLM	1.006 (0.988, 1.024)	1.019 (0.999, 1.039)
	Leroux	1.007 (0.991, 1.024)	**1.019 (1.002, 1.041)**
	OS	1.006 (0.998, 1.015)	**1.021 (1.011, 1.030)**

Employment	GLM	1.010 (0.990, 1.030)	**1.020 (1.000, 1.040)**
	Leroux	1.015 (0.998, 1.033)	**1.025 (1.007, 1.044)**
	OS	**1.014 (1.006, 1.023)**	**1.025 (1.016, 1.036)**

Housing	GLM	0.992 (0.973, 1.012)	0.989 (0.968, 1.011)
	Leroux	0.990 (0.971, 1.009)	0.980 (0.959, 1.002)
	OS	0.992 (0.983, 1.002)	**0.987 (0.977, 0.997)**

Income	GLM	1.003 (0.985, 1.021)	1.010 (0.990, 1.030)
	Leroux	1.008 (0.992, 1.108)	1.012 (0.995, 1.030)
	OS	1.007 (0.998, 1.015)	**1.013 (1.004, 1.023)**

SIMD	GLM	1.007 (0.989, 1.025)	1.017 (0.997, 1.037)
	Leroux	1.013 (0.997, 1.030)	**1.021 (1.003, 1.040)**
	OS	**1.011 (1.003, 1.020)**	**1.021 (1.011, 1.030)**


**Table 3:** Model fit for each of the 42 models, measured by the Deviance Information Criterion (DIC), the effective number of parameters (*p_D_*), and the root mean square error (RMSE).

Deprivation	Model	DIC (*p_D_*)		RMSE	
		Fusion	DEFRA	Fusion	DEFRA
Access	GLM	20219 (2)	20182 (2)	13.560	13.519
	Leroux	13797 (1508)	13799 (1507)	2.518	2.525
	OS	19130 (76)	19115 (74)	12.614	12.604

Crime	GLM	20017 (2)	19967 (2)	13.471	13.429
	Leroux	13793 (1498)	13791 (1497)	2.511	2.510
	OS	19222 (67)	19201 (66)	12.707	12.697

Education	GLM	18240 (2)	18224 (2)	12.742	12.724
	Leroux	13601 (1369)	13600 (1367)	2.687	2.688
	OS	17964 (62)	17942 (62)	12.336	12.319

Employment	GLM	18373 (2)	18359 (2)	12.811	12.812
	Leroux	13600 (1378)	13597 (1377)	2.655	2.658
	OS	18010 (66)	17996 (65)	12.323	12.318

Housing	GLM	19107 (2)	19106 (2)	12.989	12.993
	Leroux	13737 (1451)	13736 (1450)	2.522	2.522
	OS	18336 (69)	18352 (69)	12.302	12.323

Income	GLM	18139 (2)	18135 (2)	12.638	12.623
	Leroux	13589 (1362)	13589 (1362)	2.693	2.692
	OS	17743 (66)	17729 (60)	12.128	12.129

SIMD	GLM	18277 (2)	18267 (2)	12.701	12.694
	Leroux	13609 (1374)	13606 (1373)	2.672	2.670
	OS	17900 (62)	17898 (64)	12.242	12.240

